# Photocatalytic deposition of noble metals on 0D, 1D, and 2D TiO_2_ structures: a review

**DOI:** 10.1039/d4na00623b

**Published:** 2024-11-06

**Authors:** Salih Veziroglu, Josiah Shondo, Tim Tjardts, Tamim B. Sarwar, Ayse Sünbül, Yogendra Kumar Mishra, Franz Faupel, Oral Cenk Aktas

**Affiliations:** a Chair for Multicomponent Materials, Department of Materials Science, Faculty of Engineering, Kiel University Kaiserstr. 2 24143 Kiel Germany sve@tf.uni-kiel.de oca@tf.uni-kiel.de; b Kiel Nano, Surface and Interface Science KiNSIS, Kiel University Christian Albrechts-Platz 4 24118 Kiel Germany; c Fraunhofer Institute for Photonic Microsystems IPMS, Center Nanoelectronic Technologies CNT An der Bartlake 5 Dresden 01109 Germany; d Mads Clausen Institute, NanoSYD, University of Southern Denmark Alsion 2 6400 Sønderborg Denmark

## Abstract

In recent years, extensive research on noble metal–TiO_2_ nanocomposites has demonstrated their crucial role in various applications such as water splitting, self-cleaning, CO_2_ reduction, and wastewater treatment. The structure of the noble metal–TiO_2_ nanocomposites is critical in determining their photocatalytic properties. Numerous studies in the literature describe the preparation of these nanocomposites with various shapes and sizes to achieve tunable photocatalytic performance. However, achieving a stable coupling between the noble metal and the TiO_2_ surface remains a challenge for long-term use. Photocatalytic deposition is one of the most promising approaches to obtain well-defined noble metal structures on TiO_2_ surfaces with strong adhesion. Noble metal nanoparticles (NPs) can be quickly grown on the TiO_2_ surface under light exposure. However, various parameters such as the pH, temperature, precursor, and electron sacrificial agent affect the size and distribution of the deposited particles. In this review article, we look at the critical parameters that influence the photocatalytic deposition of noble metals on major TiO_2_ morphologies, classified as 0D (NPs and nanocrystals), 1D (nanotubes and nanowires), and 2D (thin films).

## Abstract 2

In recent years, extensive research on noble metal–TiO_2_ nanocomposites has demonstrated their crucial role in various applications such as water splitting, self-cleaning, CO_2_ reduction, and wastewater treatment.

## Graphical abstract

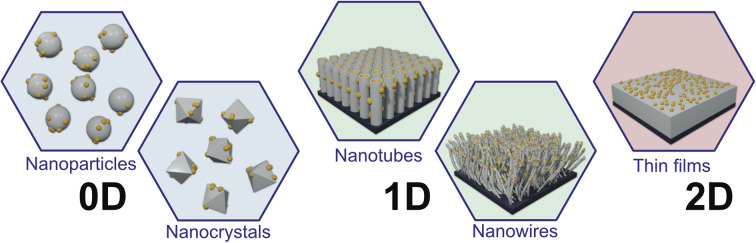

## 1 Introduction

**Fig. 1:**
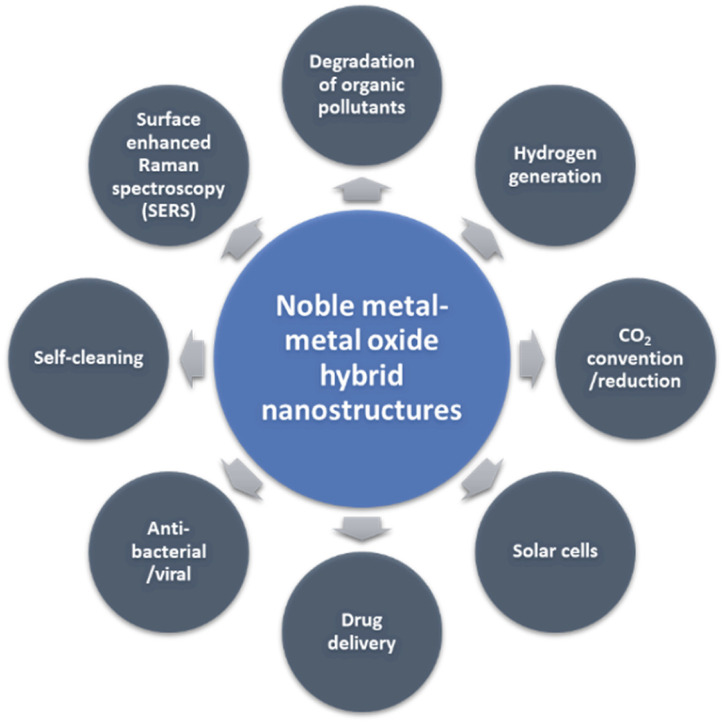
General applications of noble metal–metal oxide hybrid nanostructures.

Over the last few decades, nanomaterials have attracted exceptional research interest due to their unique physical and chemical properties, mainly arising from their high surface area (enormous surface-to-volume ratios) and nanoscale size.^1^ Among the different kinds of nanomaterials, noble metal (Au, Ag, Pt, Pd, as well as the Au/Ag alloy) and metal oxide (TiO_2_, ZnO, Cu_2_O, and CeO_2_) nanocomposites with well-defined structures and properties have gained significant attention for many applications, especially in photocatalysis^2^ (degradation of organic pollutants,^3^ photocatalytic hydrogen generation, photocatalytic CO_2_ reduction, *etc.*), solar cells,^4^ biomedicine^5^ and surface-enhanced Raman spectroscopy (SERS) (Fig. 1).^6,7^ The effectiveness of these hybrid nanocomposites depends highly on their size,^8^ shape,^4^ and structure-dependent properties (*e.g.*, surface and interface chemistry).^9^ Therefore, the rational design (new structures with specific functionality), well-controlled synthesis and detailed characterization, and a better understanding of structure–property relationships are crucial to the functionality of the nanocomposites and their use in real-world applications.^10^

In the literature, several methods exist to prepare functional noble metal–metal oxide nanostructures, including impregnation,^11^ chemical vapor deposition,^12^ solvothermal synthesis,^13^ electrodeposition,^14^ atomic-layer deposition (ALD),^15^ sputtering,^16^ physical mixing,^12^ and green synthesis^17^ (Table 1). Among these methods, photocatalytic deposition (photodeposition or photoinduced reduction) is attractive due to its simplicity. It requires only illumination or irradiation to produce well-defined metal nanoparticles (NPs) in a simple reactor system without recourse to high temperature and cost. However, it requires some specific parameters, such as light intensity, to control the structure, size, surface coverage and oxidation state of the deposited NPs.^18–20^

**Table 1:** Comparison of noble metal deposition methods, highlighting their advantages and disadvantages

Method	Advantages	Disadvantages
Photodeposition	• Mild reaction conditions	• Requires specific light conditions (*e.g.*, UV)
	• Low cost and simple setup	• Limited to certain metals and substrates
	• Green and safe process	• May have lower deposition rates compared to other methods
	• Control over spatial distribution of nanoparticles	
	• Potential for various applications (photocatalysis, electrocatalysis, *etc*.)	
Simple reduction process	• Low-cost and straightforward method	• Often requires additional reducing agents unless spontaneous reduction is used
	• Can be performed under ambient conditions	• Control over particle size can be limited
	• Minimal equipment required	
Chemical vapor deposition (CVD)	• High purity and uniformity of films	• High temperatures required
	• Suitable for large-scale production	• Complex equipment setup
	• Good control over thickness	• Potentially hazardous chemicals involved
Sputtering	• Versatile for different materials	• Requires vacuum conditions
	• Good adhesion to substrates	• Can lead to non-uniform coatings
	• Can produce thin films with controlled thickness	• Equipment can be expensive
Electrochemical deposition	• Low cost and simple setup	• Limited to conductive substrates
	• Good control over morphology and composition	• May require post-deposition treatments for optimal properties
	• Can be done at room temperature	
Laser ablation	• High control over particle size and shape	• Expensive equipment needed
	• Fast processing times	• Limited scalability for mass production
	• Minimal contamination risk	
Atomic layer deposition (ALD)	• Atomic-level control over thickness and composition	• Slow deposition rates
	• Excellent uniformity on complex surfaces	• High cost of precursors and equipment

In recent years, photodeposition has emerged as an ideal method in photocatalysis, with applications ranging from the degradation of organic pollutants and photocatalytic hydrogen generation to self-cleaning surfaces and photocatalytic CO_2_ reduction. Despite its widespread use, the precise origins of the term “photodeposition” remain unclear. One of the earliest recorded uses of this term can be traced back to a study by Sadowsky and Payne in 1957.^21^ The term was used to explain the deposition of a phosphor color screen for a cathode ray tube. Photodeposition involves irradiating a semiconductor (slurry, paste, powder, or a thin film) in metal salt solution resulting in metal nanoparticle deposition on the semiconductor surface. Photoexcited electrons from the semiconductor reduce the metal cations with suitable redox potential, resulting in the formation of metal nanoparticles on the semiconductor surface.^22,23^

Generally, in semiconductors, the magnitude of the band gap determines the electrical, electronic, and optical properties of the semiconductors. The band gap also defines the wavelength sensitivity of the semiconductors to light exposure. Photoexcitation with light of energy larger than the band gap energy promotes an electron from the valence band to the conduction band, creating an electronic vacancy or “hole” (h^+^) in the valence band (see eqn (1)).^24^1
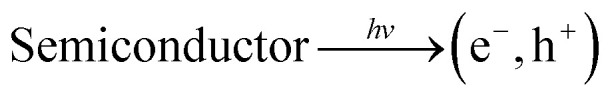


**Fig. 2:**
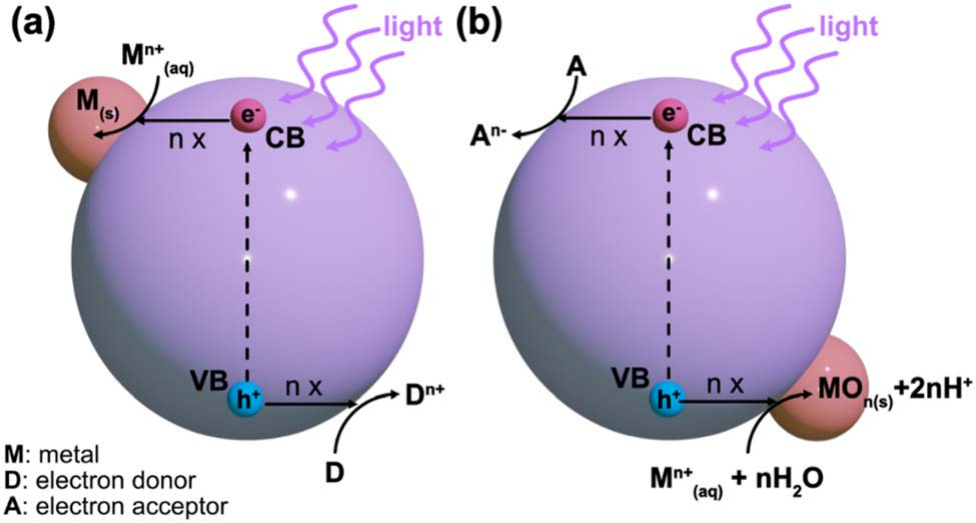
Schematic illustration of (a) reductive and (b) oxidative photodeposition processes.

Typically, the energy level at the bottom of the conduction band determines the reducing ability of the photoelectrons, while the energy level at the top of the valence band determines the oxidizing ability of the photogenerated holes.^25^ Both of these species can be utilized in photocatalytic redox reactions. Normally, the conduction band electrons facilitate reduction reactions with the surrounding chemical species, while the valence band holes provide electron acceptors for photocatalytic oxidation.^23^ This understanding is critical for designing metal-supported catalysts *via* the photodeposition technique. For a successful photocatalytic reduction, the photocatalyst's conduction band edge should be more negative than the reduction reaction's standard potential on the NHE scale.^23^ Conversely, for successful photocatalytic oxidation, the valence band edge must be more positive than the corresponding reduction's standard potential. Photodeposition relies on photocatalytic redox reactions with precursor species, which is schematically shown in Fig. 2. In the photodeposition of metals, the corresponding generalized reduction reaction from a metal precursor ion M^*n*+^ reduced by *n* conduction band electrons is given in eqn (2).^23^

2 M_(aq)_^*n*+^ + *n*e^−^ → M_(s)_

Alternatively, in an oxidative counterpart reaction with *n* valence band holes and water, a metal oxide species is formed, as shown in eqn (3)^23^

3 M_(aq)_^*n*+^ + *n*h^+^ + *n*H_2_O + MO_*n*_(s) + 2*n*H^+^

When a metal particle with a higher work function is in contact with an n-type semiconductor, electrons transfer from the semiconductor to the metal particle.^26,27^ Consequently, at the metal–semiconductor interface, a rectifying Schottky barrier is formed.^28^ This barrier facilitates charge separation by reducing the electron–hole recombination, thereby enhancing the photocatalytic activity of materials like TiO_2_.^29^ Because of the improved separation of electrons and holes, metal deposition on the semiconductor surface enhances photocatalytic reactions by hastening the transfer of electrons to reactants, such as dissolved oxygen molecules.^30^ The prediction of Schottky barrier formation and overall band structure typically relies on literature-reported or experimentally determined work functions as a starting point. However, the work function of metal oxide semiconductors like TiO_2_ is highly sensitive to experimental conditions, leading to a wide range of reported values.^31,32^ Moreover, the work functions can vary depending on factors such as crystallinity, particle geometry^33,34^ and the surrounding environment.^32^ This makes accurate predictions of band structure and Schottky barrier formation challenging as these properties are influenced by the crystal structure, morphology and the operational environment.

Among many semiconductor materials, TiO_2_ is one of the most suitable candidates for industrial applications due to its photoactivity, high chemical stability, eco-friendliness, and cost effectiveness. However, it has two drawbacks restricting its photocatalytic applications: limited photoresponse range (*λ* < 380 nm) and low quantum yields at longer wavelengths (*λ* > 400 nm). The quantum yield increases upon modification of the TiO_2_ surface with noble metals such as Au, Ag, Pt, and Pd due to the formation of a Schottky contact.^20^ Moreover, when the TiO_2_ surface is modified with noble metals, it can lead to a shift in the absorption edge of the TiO_2_ to the vis region (narrowing band gap). Also, localized surface plasmonic activity of the noble metals boosts the excitation of active charge carriers.^35^ Due to these synergetic effects of noble metals and TiO_2_, an enhancement in the photocatalytic activity is achieved, providing an excellent opportunity for industrial use under solar light illumination.^36^ Hence, photocatalytic deposition of noble metals and metal oxides on TiO_2_ has gained more attention in recent years because of its applications in photocatalytic solar fuel synthesis,^37^ wastewater treatment,^38^ air purification,^24^ self-cleaning,^39,40^ and biomedical applications.^41^

Photoinduced redox reactions at the surface of TiO_2_ have been known since the early 20th century. In 1920/21, Carl Renz reported observations of the darkening effect of TiO_2_ when exposed to sunlight.^42^ Later, it was shown that photoreactions with the surrounding medium, including exposure of TiO_2_ to light in the presence of silver nitrate (AgNO_3_), resulted in the reduction of AgNO_3_ to metallic Ag (deposition of Ag particles).^43^ A few decades later, Clark and Vondjidis reported infrared spectroscopy studies of TiO_2_ NPs mixed with AgNO_3_. They showed that AgNO_3_ was reduced to metallic Ag after UV irradiation of the mixture.^44^ However, the study, which was reported by Kraeutler and Bard in 1978, sparked much research interest in the photodeposition process.^45^ In this report, platinum (Pt) was photodeposited on TiO_2_ (anatase) by irradiating a paste containing anatase powder, hexachloroplatinic acid (H_2_PtCl_6_), hydrochloric acid (HCl), sodium carbonate (Na_2_CO_3_), and acetic acid (CH_3_COOH) as an electron scavenger. During the reaction process, the system was purged with nitrogen to remove O_2_ and CO_2_, and the temperature was increased to 55 °C. Finally, it was shown that well-dispersed Pt NPs could be obtained *via* photodeposition.

**Fig. 3:**
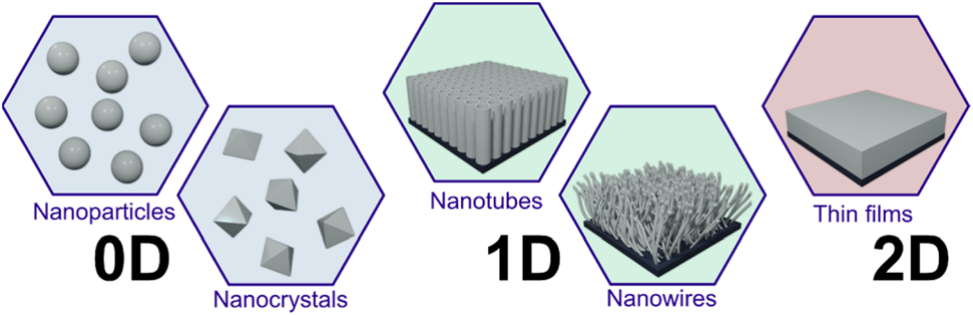
Schematic illustration of the most used TiO_2_ morphologies (0D: NPs and nanocrystals; 1D: nanotubes and nanowires; and 2D: thin films).

The photodeposition process has been known for more than 55 years in the scientific community. However, it is still a challenge to understand all process parameters to obtain well-desired structures for specific applications since several parameters need to be considered to allow the photodeposition process to occur on the semiconductor surface, such as the sacrificial reagent,^46^ pH,^47^ temperature,^48^ metal precursor,^49^ light exposure time^50^ and intensity,^51^ and absence or presence of oxygen in the media.^23^ To date, many research and review articles have concentrated on the effect of such photodeposition parameters, especially using TiO_2_.^23,52–54^ Only a few studies have focused on investigating the impact of the morphology of TiO_2_ on the photodeposition process, unlike other deposition parameters. The morphology directly influences the photodeposition process by affecting the specific/active surface area, availability of charge carriers, pore structure, crystalline phase, and exposed surface facet.^55,56^ Therefore, the morphology differences need to be considered as one of the key points to achieve well-desired photodeposited nanostructures on the TiO_2_ surface. As depicted in Fig. 3, variable morphologies of TiO_2_ are categorized as 0D: NPs and nanocrystals; 1D: nanotubes and nanowires; and 2D: thin films.

So far, numerous research articles have reported on the preparation of noble metals on TiO_2_ surfaces *via* photodeposition, highlighting the advantages of this approach.^46,52,57,58^ Additionally, excellent reviews have been published in the past few years on the photodeposition process that focused on the methods, mechanisms, and applications of photodeposition.^23,59^ In most cases, the authors have focused on the effect of deposition parameters, which include the sacrificial electron donors, pH, precursor, temperature, as well as the absence and presence of oxygen.^23,59,60^ However, a comprehensive review analyzing the role of various TiO_2_ morphologies in the catalytic activity and photodeposition processes is still lacking, hindering further advancement in photocatalytic research. Therefore, in this review article, we provide an overview of the recent research progress on the photodeposition of noble metals on different TiO_2_ morphologies such as 0D: NPs and nanocrystals; 1D: nanotubes and nanowires; and 2D: thin films. The following sections can give researchers different perspectives on the photodeposition process based on used materials and systems. Additionally, future perspectives on preparing future metal–semiconductor systems *via* photodeposition will be presented and discussed.

## 2 Photocatalytic deposition of noble metal nanoparticles on different TiO_2_ morphologies

### 2.1. 0D structures

#### 2.1.1. TiO_2_ nanoparticles

0D structures refer to materials with all dimensions confined to the nanoscale, typically below 100 nanometers. These structures often take the form of nanoparticles, which can exhibit unique physical and chemical properties due to their small size and high surface-to-volume ratio. Common examples of 0D nanomaterials include nanoparticles, quantum dots, and fullerenes. As is known, Degussa P25 (*ca.* 70–80% anatase and 30–20% rutile) is the brand name of TiO_2_ NPs widely used in photocatalytic reactions due to its relatively high photoreactivity. It is difficult to find a photocatalyst that has an activity that is higher than the activity of P25 TiO_2_.^61^ Therefore, P25 TiO_2_ has been used as a de facto standard titania photocatalyst. Hence, since the 1900s, more than a thousand articles have been reported on the P25 TiO_2_ photocatalytic reactions.^62^ The phase composition of Degussa Evonik P25 is not clearly defined, as the exact ratios of anatase and rutile can vary depending on production methods and conditions.^63^ However, Table 2 reveals other essential properties of P25 TiO_2_ based on the information supplied by Evonik (Evonik Research Efficiency GmbH, 2020).^64^

**Table 2:** Summary of specific properties of P25 TiO_2_ (ref. 64)

Properties	Units	Value
Specific surface area	m^2^ g^−1^	35–65
Approximate particle size^64^	Nm	20
pH value in 4% dispersion	—	3.5–4.5
Tapped density	g L^−1^	100–180

Research groups have provided detailed insights into the phase structure of P25 TiO_2_. It has been established that P25 TiO_2_ powder comprises multiphasic TiO_2_ nanoparticles (NPs), consisting of approximately 80% anatase and 20% rutile in the crystalline phase. Additionally, an amorphous phase has been identified through X-ray diffraction (XRD) analysis and transmission electron microscopy (TEM) measurements.^64^ It has been determined that the anatase to rutile ratio changes from batch to batch and should be somewhere between 70 : 30 and 80 : 20.^65^

P25 TiO_2_ has been frequently used as a substrate material for the photodeposition of noble metals, among other TiO_2_ NPs.^30,46,58,67,68^ Sclafani and Herrmann first demonstrated the growth of noble metals on P25 TiO_2_ in 1998.^69^ They reported the effect of grown Ag nanoparticles on the photocatalytic activity of TiO_2_, specifically rutile and anatase phases in both organic and aqueous media. Ag–P25 TiO_2_ samples were prepared *via* photocatalytic deposition by irradiating an aqueous AgNO_3_ solution with a 125 W high-pressure mercury lamp, using a 300 nm optical filter.^69^ Furthermore, the authors investigated the effect of deposited noble metals (Ag and Pt) as both single and bimetallic systems on the distinctive phases of P25. Notably, in the anatase phase, Pt selectively grows atop pre-existing Ag particles, showing 100% selectivity. Whereas, in the rutile phase, Pt can grow both on Ag particles and directly on the rutile surface. Additionally, the study showed that Ag deposits are beneficial for rutile activity but has detrimental effect on anatase activity.^69^

Over the past few decades, the photodeposition of noble metals on commercial P25 TiO_2_ has been investigated to improve control over the particle size, distribution, and morphology for specific applications.^58,66^ In this regard, Bhardwaj *et al.* studied the effects of different wt% of Ag loadings on commercial P25 TiO_2_ and different deposition times (30–90 min) on the structural, optical, and photocatalytic properties of Ag/TiO_2_ nanocomposites. The study showed that increasing the loading amount and UV irradiation time during photodeposition enhanced the plasmonic response, resulting in uniform nanostructure growth and regular distribution of Ag NPs.^58^

**Fig. 4:**
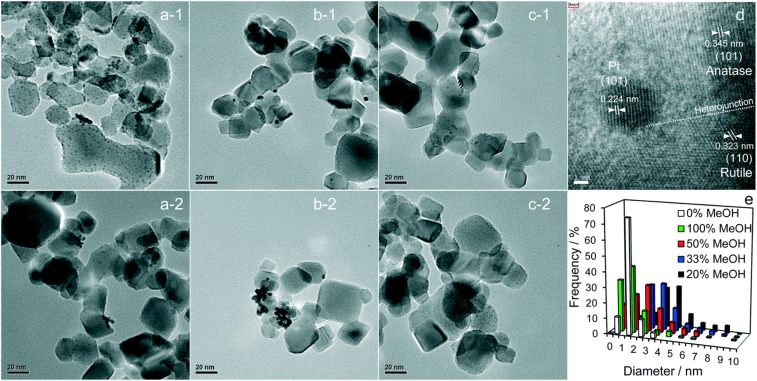
Representative bright-field HRTEM images of (a-1 and a-2) 100%, (b-1 and b-2) 20%, and (c-1 and c-2) 0% MeOH with 1 wt% Pt/TiO_2_, and (d) an individual Pt metal nanoparticle at the interface between anatase and rutile crystallites. (e) Particle size distributions as a function of methanol concentration during PD. Adapted with permission from ref. 66. Copyright (2016) Royal Society of Chemistry (RSC).

Pt is the most effective metal catalyst in conventional thermal oxidation processes among other noble metals. In particular, the loading of Pt onto the P25 TiO_2_ surface increases the efficiency of charge separation and promotes the amount of absorbed organic compound on the surface. Platinum (Pt) functions as an electron sink through the formation of a charge-transfer complex with TiO_2_. The catalytic properties of Pt are particularly influenced by several factors, including the nanoparticle size, dispersion, morphology, and oxidation state.^52^ Jiang *et al.*^66^ showed that methanol is critical for controlling Pt formation on P25 TiO_2_ during photodeposition. Their study investigated the role of methanol in the fabrication of Pt loaded on P25 TiO_2_ for photocatalytic H_2_ generation. Pt was deposited on TiO_2_ using chloroplatinic acid (H_2_PtCl_6_) as a precursor in an aqueous solution with various methanol concentrations under 300 W Xe lamp illumination with an intensity of 124.6 mW cm^−2^.^66^ It was found that the resulting H_2_ production rate increased linearly with the vol% of methanol used in the precursor solution. Fig. 4 shows bright-field high-resolution transmission electron microscopy (HRTEM) images of the resulting Pt-loaded P25 TiO_2_ NPs and the corresponding particle distributions. The dark spots in the bright-field HRTEM images can be associated with the Pt loading due to *Z* contrast. Fig. 4a-1 and a-2 exhibit the Pt-loaded P25 TiO_2_ surfaces when 100 vol% methanol was used during the deposition.

When considering *Z* contrast, the small dark spots on the grey particles can be associated with highly dispersed small Pt NPs. Fig. 4b-1 and b-2 show the resulting Pt-loaded P25 TiO_2_ when 20 vol% methanol was used during deposition. Comparing these images to those of a 100 vol% sample, it was observed that the overall dispersion of the Pt NPs has decreased, and the overall size of the Pt NPs increased. For the samples prepared in a 0 vol% methanol solution, highly dispersed dark spots can be seen in the resulting HRTEM images shown in Fig. 4c-1 and c-2. Here, the dispersion and size of the samples appear comparable to those of the 100% vol sample. Fig. 4d depicts a Pt nanoparticle on the P25 TiO_2_ surface. Based on the structure of the crystallites present, it can be concluded that the Pt NPs grow at the interface between anatase and rutile crystallites, indicating no preferential substrate crystal phase. In fact, Jiang *et al.*^66^ reported that the Pt NPs were deposited randomly across the anatase and rutile surfaces of P25 TiO_2_. The particle distribution of Pt particles can be extracted from TEM images of particles with different vol% samples used during sample preparation, as shown in Fig. 4e. It can be observed that both 100 vol% and 0 vol% methanol lead to highly dispersed Pt NPs (mostly <2.5 nm), whereas 20 vol% methanol leads to larger Pt NPs (some >4 nm). Upon an increase in methanol from 20 vol% up to 100 vol%, a decrease in the overall particle size and an increase in the dispersion could be observed. This is consistent with the TEM images discussed above. The decrease in particle size and the increase in dispersion can be attributed to methanol acting as a hole scavenger, suppressing recombination in TiO_2_ during the photodeposition and leading to electron-rich surfaces where metallic Pt can be deposited. At 0 vol%, non-metallic Pt compounds like PtO_2_ and PtCl lead to high dispersion. Still, an increase in methanol concentration causes metallic Pt to be produced more frequently, initially leading to large metallic Pt NPs at low concentrations. The Pt loading shows more dispersion and a decrease in the size of particles as the methanol concentration increases. This leads to the conclusion that the photodeposition of Pt on P25 TiO_2_ methanol drives both Pt reduction and its subsequent redispersion.

Over the years, numerous studies have been published based on the photodeposition of noble metals on P25 TiO_2_ NPs for various applications (Table 3).^22,30,46,53,57,58,66–68,70–73^ Investigation of these data concludes that mercury lamps and sometimes xenon lamps were preferentially used to perform the photodeposition reactions. Usually, an aqueous AgNO_3_ solution is used as a precursor for the photodeposition of Ag NPs. However, different sacrificial agents can be used for specific purposes, such as varying particle sizes and surface coverages. Chlorine-based precursors such as H_2_PtCl_6_ and HAuCl_4_ solutions are commonly used to prepare Pt and Au NPs, respectively. These chlorine-based compounds are readily available and have high solubility in water.

**Table 3:** Overview of different noble metal NPs photodeposited on TiO_2_ NPs for various applications (the wavelength refers to the maximum wavelength of the emission spectrum of the respective light source)

Deposited noble metal	UV source and wavelength (*λ*)	Precursor molecule; additional agent	Possible applications	Reported in
Ag	Mercury arc lamp, 125 W 10.4 mW cm^−2^, *λ* not given	AgNO_3_; isopropanol	Photodegradation of salicylic acid	Bhardwaj *et al.*^58^
Ag	Xenon lamp, 300 W, *λ* not given	AgNO_3_; sodium hydrogen carbonate	Photocatalytic reduction of CO_2_ with water	Hammad *et al.*^53^
Ag	Mercury lamp, 500 W, *λ* = 365 nm	AgNO_3_; methanol	Not given	Ma *et al.*^70^
Ag	UV-A lamp, 40 W, *λ* = 350–400 nm	[Ag(S_2_O_3_)_2_]^3^ in radiographic wastewater	Bacterial disinfection	Wahyuni *et al.*^71^
Ag and Pt	Mercury lamp, 200 W, *λ* = 365 nm	AgNO_3_	Not given	Taing *et al.*^57^
		K_2_PtCl_4_		
		Trisodium citrate		
Pd	Low-pressure mercury lamp, 250 W m^−2^, *λ* not given	PdCl_2_; methanol	Organic photodegradation reactions	Dadsetan *et al.*^68^
Pd	Low-pressure mercury lamp, 250 W m^−2^, *λ* not given	PdCl_2_; methanol, ethanol, ethylene glycol, and 1-isopropanol	Not given	Dadsetan *et al.*^46^
Bimetallic Pd–Au	Mercury lamp, 6 W, *λ* = 254 nm	Pd(C_5_H_7_O_2_)_2_	Selective oxidative esterification of methanol to form methyl formate	Colmenares *et al.*^72^
		HAuCl_4_·3H_2_O		
		Acetonitrile		
Pt	Super-pressure mercury lamp, 30 mW cm^−2^, *λ* = 240–500 nm	H_2_PtCl_6_; methanol	Photocatalytic H_2_ evolution	Haselmann *et al.*^30^
Pt	Xenon lamp, 300 W, *λ* not given	H_2_PtCl_6_; methanol	Photocatalytic H_2_ evolution	Jiang *et al.*^66^
Au and Pt	Mercury lamp, 0.15 W m^−2^, *λ* = 365 nm	HAuCl_4_	Not given	Galeano *et al.*^73^
		H_2_PtCl_6_		
		Isopropanol		
Au, Pt, and Rh	Mercury–xenon lamp, 200 W, *λ* not given	AuCl_3_ (Au_2_Cl_6_)	Not given	Ohyama *et al.*^22^
		H_2_PtCl_6_·6H_2_O		
		RhCl_3_		
		Methanol		
Au, Pt, Rh, Pd, and Ag	High-pressure mercury arc lamp, *λ* > 300 nm	HAuCl_4_	Water splitting without using any reagents under irradiation of visible light	Tanaka *et al.*^67^
		Colloidal Au nanoparticles		
		Oxalic acid		
		Other precursors not given		

#### 2.1.2. TiO_2_ nanocrystals

**Fig. 5:**
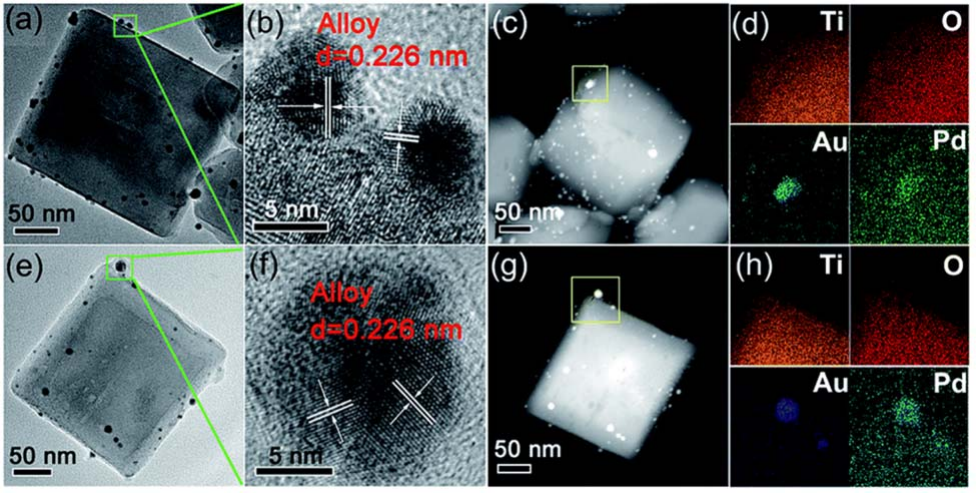
Detailed morphology and composition analysis of two representative samples: (a–d) CR-Au_1_Pd_1_ and (e–h) PD-Au_1_Pd_1_. (a and e) TEM images, (b and f) HR-TEM images, (c and g) HAADF-STEM images, and (d and h) corresponding EDX mapping of Ti, O, Au, and Pd, respectively. Adapted with permission from ref. 89. Copyright (2018) Royal Society of Chemistry (RSC).

Nanocrystals (NCs) are aggregates of atoms that combine into a “cluster” and are less than 1 μm in size. Typical sizes range between 10 and 400 nm.^74^ TiO_2_ nanocrystals have been extensively used in materials research, chemical engineering, and as quantum dots for biological imaging.^75,76^ Synthesizing TiO_2_ NCs with a high surface area is vital to maximizing the properties of the nanocrystals. Essentially, TiO_2_ has three distinct crystallographic phases (anatase, rutile, and brookite) of which anatase and rutile (thermodynamically more stable forms) in the ratio of 70 : 30 have been utilized as excellent photocatalysts for photodecomposition of organic pollutants and solar-energy conversion due to their high photoactivity.^77,78^ In general, TiO_2_ has been shown to possess an anatase tetragonal bipyramidal polymorphic structure that mainly exposes the (101) planes, with a small percentage of the (001) facet based on Wulff construction.^79^ Typically, anatase TiO_2_ NPs have been mainly synthesized by wet chemical synthesis methods such as sol–gel,^80–83^ micelle/reverse micelle,^84^ and hydrothermal methods.^85^ The sol–gel method proceeds with the hydrolysis of titanium(iv) alkoxide, followed by condensation.^86^ In the micelle/reverse micelle method, TiO_2_ nanocrystals are obtained by controlled micelle or reverse micelle formation by hydrolysis of a titanium alkoxide precursor and then annealing the sample to induce crystallinity.^84^ In the hydrothermal method, dehydration of an aqueous solution of titanium salt at high temperatures provides nanocrystals.^87^ Liu *et al.*^88^ utilized a two-step hydrothermal method to synthesize well-defined truncated tetragonal bipyramidal TiO_2_ NCs having (001) facets exposed on the top/bottom surface and (101) on the side surface. Subsequently, Au–Pd alloy NPs with a fixed Au to Pd atomic ratio of 1 : 1 were immobilized on the pristine TiO_2_ NCs *via* photodeposition that resulted in PD-Au_1_Pd_1_ composition. Similarly, a reference sample of Au : Pd in a 1:1 ratio was prepared by ascorbic acid (AA) induced chemical reduction (*i.e.*, CR-Au_1_Pd_1_). By TEM analysis (Fig. 5a and e), Chen *et al.* showed that CR-Au_1_Pd_1_ possessed some Au–Pd NPs of darker contrast evenly anchored on the (101) and (001) facets of the TiO_2_ NCs, while the Au–Pd NPs of PD-Au_1_Pd_1_ were specifically deposited on the (101) facets of the TiO_2_ NCs. HR-TEM images of these NPs on CR-Au_1_Pd_1_ and PD-Au_1_Pd_1_ revealed clear lattice fringes with an interlayer spacing of 0.226 nm, corresponding to the (111) planes of a face-centered cubic (FCC) metal (Fig. 5b and f).^90,91^

**Fig. 6:**
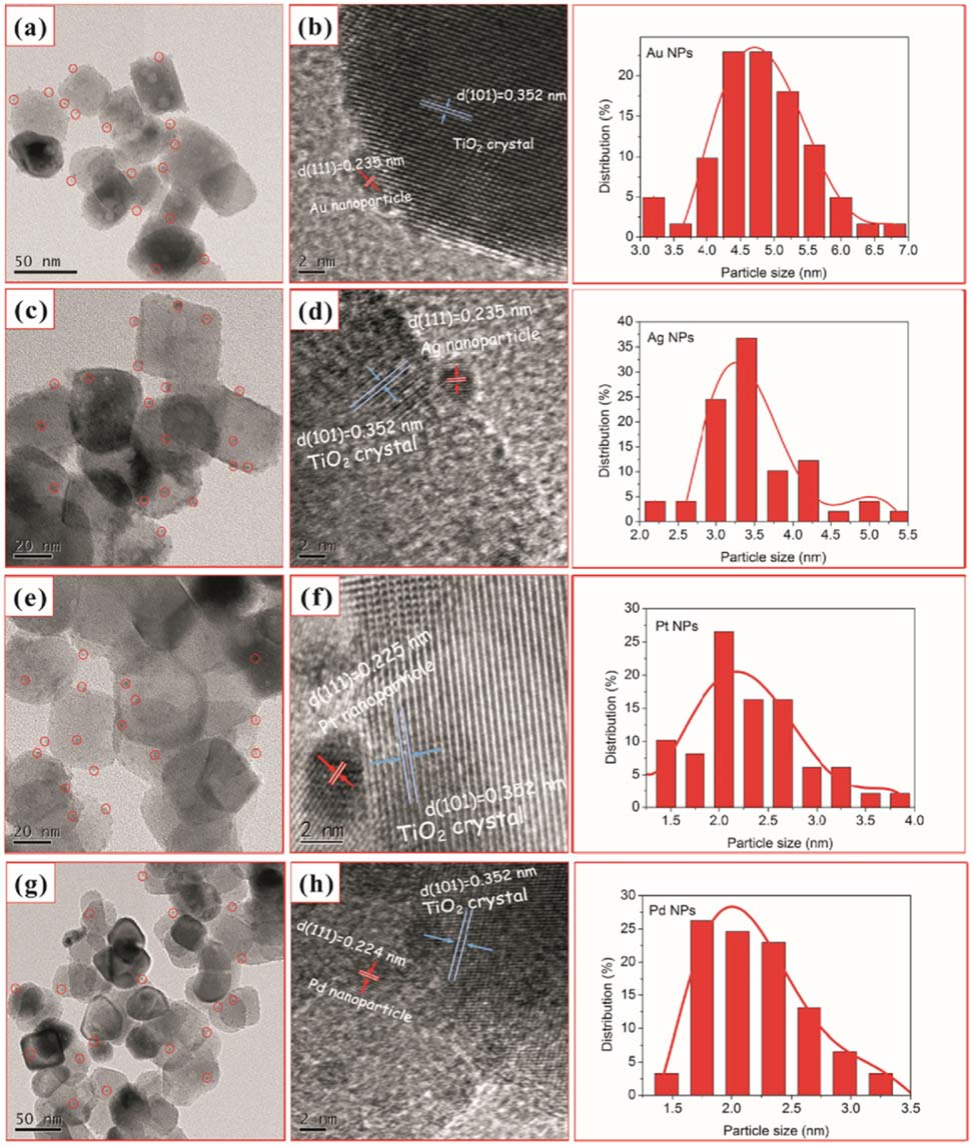
TEM and HRTEM images of TiO_2_–H after loading with 2 wt% Au (a and b), Ag (c and d), Pt (e and f), and Pd (g and h) and (right) nanoparticle size distribution for the corresponding M–TiO_2_ (M = Au, Ag, Pt, and Pd). Adapted with permission from ref. 92. Copyright (2017) Elsevier.

Similarly, Chen *et al.*^92^ used one-step *in situ* photodeposition to deposit different noble metals such as gold, silver, platinum, and palladium on TiO_2_ nanocrystals (Fig. 6). The photocatalytic activities of M–TiO_2_ (M = Au, Ag, Pt, and Pd) samples were evaluated by the photocatalytic selective oxidation of benzyl alcohol. The noble metals were supported on hollow TiO_2_ nanocrystals *via* the *in situ* photodeposition method.

Here, the deposition of Au, Ag, Pt, and Pd was achieved using chloroauric acid (HAuCl_4_), silver nitrate (AgNO_3_), hexachloroplatinic(iv) acid (H_2_PtCl_6_), and palladium(ii) chloride (PdCl_2_) as the precursors, respectively. The TEM and HRTEM image analyses showed 2 wt% of noble NPs loaded on TiO_2_–H (Fig. 6). The results revealed the presence of spherical noble metal NPs on the surface of the TiO_2_ support material for different M–TiO_2_ hybrid nanostructures. As illustrated by the red circles in Fig. 6 (a, c, e, and g), Au, Ag, Pt, and Pd metal NPs were observed as dark spots uniformly decorated on TiO_2_ nanocrystals. The large conglomerates of metal NPs on TiO_2_ support were not observed. Notably, the photoreduction of Pt(iv) or Pd(ii) ions formed smaller NPs (∼2 nm) as compared to that of the solution consisting of the same amount of Au(iii) or Ag(i) ions (NPs ranging from 4 to 5 nm). The larger size of Au NPs, compared to the Pd and Pt NPs, was attributed to a weaker metal support interaction in the case of Au with TiO_2_. As shown in the HRTEM images in Fig. 6 (b, d, f, and h), characteristic lattice fringes of 0.235, 0.235, 0.225, and 0.224 nm for Au, Ag, Pt, and Pd NPs were observed and indexed to the (111) plane of face-centered cubic structures.

### 2.2. 1D structures

#### 2.2.1. TiO_2_ nanotubes

An essential factor that dominates the photocatalytic performance and application of any loaded TiO_2_ or unloaded TiO_2_ photocatalyst is the effective surface area, which is significantly higher for NPs than for bulk samples. However, in applications, the NPs are either used in a suspension or immobilized by another processing step like sintering. This disadvantage is not present in TiO_2_ nanotube arrays produced by an anodization process on a metallic Ti substrate. They provide a relatively large surface area, and the prepared nanotubes are attached to a substrate that provides immobilization for photocatalytic applications.^93^ The process of anodization for producing TiO_2_ nanotubes on a Ti substrate was first reported by Zwilling *et al.* in 1999.^94^ Fluoride in the electrolyte solution of the anodization process led to the self-organization of nanotubes instead of a continuous oxide layer in the anodization process. Since then, the TiO_2_ nanotube production by using anodization was further optimized, and today, nanotubes of several 100 μm lengths and diameters between 10 nm and >200 nm can be grown by using this process.^93^

**Fig. 7:**
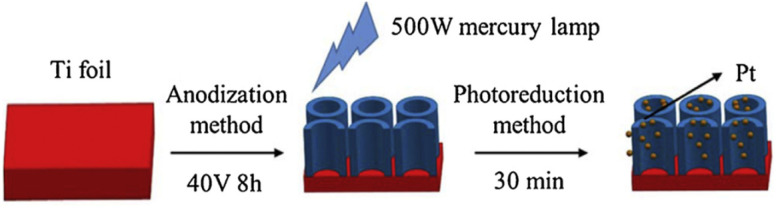
Schematic of the fabrication process of Pt/TiO_2_ NTAs. Adapted with permission from ref. 95. Copyright (2019) Elsevier.

Lv *et al.* successively constructed Pt-loaded TiO_2_ nanotube (TNT) arrays with a uniform distribution of Pt *via* photodeposition.^95^Fig. 7 schematically shows the fabrication process of Pt-loaded TiO_2_ nanotubes. First, a TiO_2_ nanotube array was produced by the well-known anodization process. A Ti foil was used as an anode, and it was oxidized in an electrolyte solution at 40 V for 8 h against a graphite cathode. The anodic oxidation of Ti led to the formation of TiO_2_ nanotube arrays.

In the following step, photodeposition of Pt on the TiO_2_ nanotubes was performed using aqueous solutions of H_2_PtCl_6_ with different concentrations and illuminating the submerged nanotube array sample with a 500 W mercury lamp for 30 minutes. XPS measurements found that the resulting Pt deposits are most likely metallic. Moreover, UV-vis absorption spectra revealed that the highest visible absorption could be achieved at an initial H_2_PtCl_6_ precursor concentration of 3 mmol L^−1^, corresponding to the highest photodegradation rate of methyl orange.

**Fig. 8:**
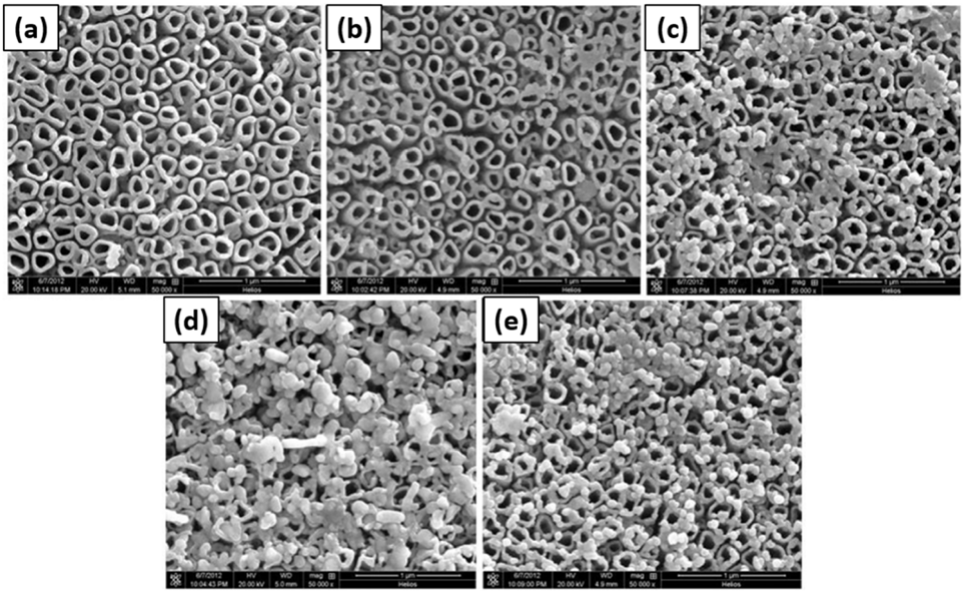
SEM images of (a) TNTs, (b) Ag-TNTs-1, (c) Ag-TNTs-2, (d) Ag-TNTs-3 and used (e) Ag-TNTs-2. Adapted with permission from ref. 96. Copyright (2016) Elsevier.

Jia *et al.* used photodeposition of Ag on TiO_2_ nanotubes to prepare photocatalysts, which were used for the photoelectrocatalytic reduction of perchlorate under a bias potential and in the presence of the hole scavenger citric acid.^96^ The TiO_2_ nanotubes were prepared by an anodization process similar to the method schematically shown in Fig. 8.

The Ag loading was carried out by the photoreduction of AgNO_3_ (volume ratio of ethanol/deionized water equal to 1 : 4) under UV illumination (light of intensity *I* = 3.5 mW cm^2^) for 30 minutes with continuous nitrogen purging. SEM images of the resulting photocatalyst are presented in Fig. 8. Bare TiO_2_ nanotubes are shown in Fig. 8a, and nearly no particles can be seen on their surface. A small number of particles are still present on the surface, which do not belong to Ag (supported by EDX analysis). Fig. 8b–d show the resulting Ag-loaded TiO_2_ nanotubes for 1, 2, and 3 g L^−1^ concentrations of initial AgNO_3,_ respectively. It was observed that the number of particles on the TiO_2_ nanotubes increased with increasing AgNO_3_ concentration. It was also noted that the average particle size increased with the increase in AgNO_3_ concentration from 9 nm to 20 nm and to 130 nm, respectively. From photoelectrocatalytic experiments, it was found that the sample shown in Fig. 8c, which was prepared with a 2 g L^−1^ AgNO_3_ solution and showing a 0.84% Ag/TiO_2_ mass ratio, exhibits the highest perchlorate reduction rate.^96^

#### 2.2.2. TiO_2_ nanowires

Compared with one-dimensional nanostructures of rods and tubes, titania nanowire (TNW) arrays have been confirmed to possess an advantage in charge separation over compact films,^97^ which is vital for photocatalysts and photoanodes. TNWs have shown to be a promising photocatalyst because of their improved structural, surface, and optoelectronic properties.^98^ Wang *et al.* and Mandal and Bhattacharyya studied Pt-modified TNWs and bare TNWs, respectively, and found 5- and 2-fold higher photocatalytic activity than P25 TiO_2_ for the degradation of methylene blue dye.^99,100^

**Fig. 9:**
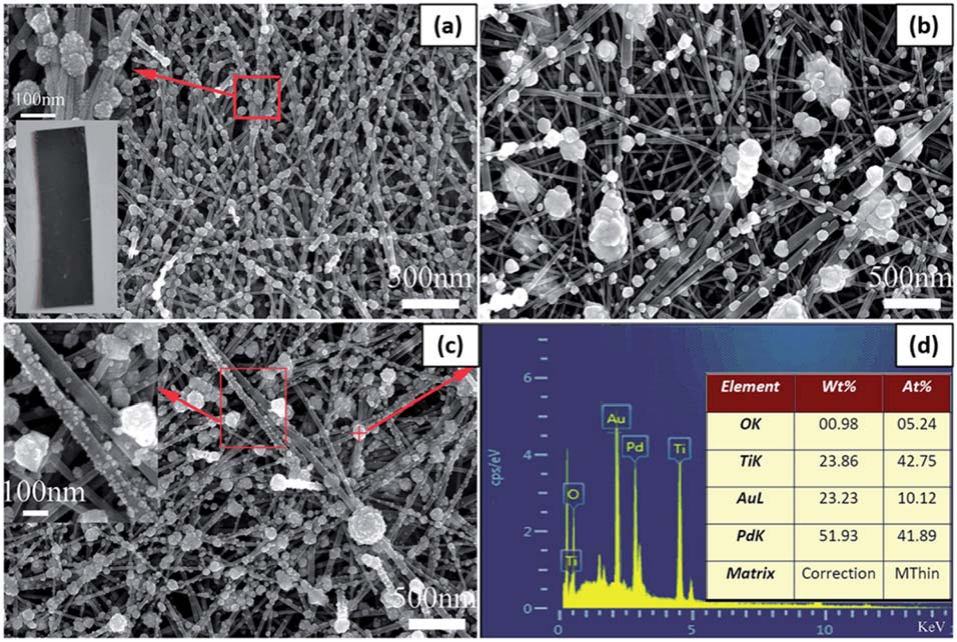
SEM pictures of (a) Pd@TiO_2_, (b) Au@TiO_2_ and (c) Pd_4_Au@TiO_2_. (d) The EDS spectrum for the particle in panel (c) marked with a red “+” symbol. The upper-left and bottom-left insets in panel (a) are the enlarged SEM image of a few NPs and the photograph of Pd@TiO_2_, respectively. The inset in panel (c) is the enlarged SEM image for the marked region with a red box. The inset in panel (d) lists the elemental distribution in Pd_4_Au@TiO_2_. Adapted with permission from ref. 101. Copyright (2016) Royal Society of Chemistry (RSC).

Ren and Liu loaded PdAu alloyed NPs on TiO_2_ nanowires *via* a one-step photochemical deposition method, and the Pd/Au ratio in alloyed NPs could be adjusted by changing the Pd^2+^/Au^3+^ ratio.^101^ Reductants and surfactants were avoided in the whole synthesis process. To synthesize PdAu alloyed NPs with different Pd/Au ratios, 16 mL of PdCl_2_ (0.5 mM) solution in ethanol was mixed with a certain amount of HAuCl_4_ (10 mM) solution in ethanol. For comparison, the same procedure was modified to deposit Pd or Au NPs on TiO_2_ nanowires with 0.5 mM PdCl_2_ or HAuCl_4_ solution, respectively. After 24 h of hydrothermal growth, TiO_2_ nanowires were densely distributed on the Ti foil. One single nanowire marked with the red arrow line was selected to estimate its length. Although its two ends exceed the measurement region, its length still reached 15 μm. As shown in the bottom-left inset in Fig. 9a, after UV irradiation for 30 min in PdCl_2_ solution, the color of the TiO_2_ nanowire film changed from milky white to black, which indicated the successful loading of Pd NPs on the TiO_2_ surface. As shown in Fig. 9a, a large amount of Pd NPs was loaded on the TiO_2_ surface, and the majority was 35–65 nm in diameter. A magnified SEM picture of a few Pd NPs indicated that a single nanoparticle on the nanowire comprised an agglomerate of many smaller NPs, as shown in the upper-left inset in Fig. 9a.

When HAuCl_4_ solution was used to deposit Au NPs under identical conditions, the as-synthesized Au nanostructures exhibited a lower density and wider size distribution than Pd NPs loaded NWs (Fig. 9b). It should be noted that their size obviously decreases along with the loading site changing from upper TiO_2_ nanowires to bottom ones. The mixed solution of PdCl_2_ and HAuCl_4_ was used to synthesize PdAu alloyed NPs on TiO_2_ nanowires. Fig. 9c shows the Pd_4_Au alloyed NPs synthesized with a Pd^2+^/Au^3+^ = 4 ratio in precursor solutions. Except for a few NPs with a diameter of about 100 nm, a large number of smaller NPs were loaded on the TiO_2_ surface, as shown in the inset in Fig. 9c. Fig. 9d shows the corresponding energy dispersive spectroscopy (EDS) spectrum of the single particle marked with the “+” symbol in Fig. 9c. Among the four elements, the O and Ti elements came from the TiO_2_ substrate, and Pd and Au came from the photodeposited NPs. The Pd/Au ratio in alloyed NPs was 4.14, which is almost the same as the Pd^2+^/Au^3+^ ratio in the precursor solution.

**Fig. 10:**
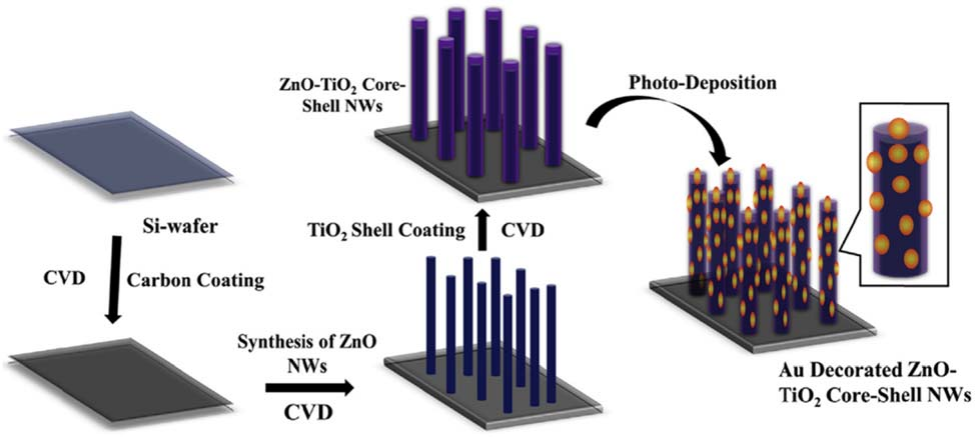
Schematic representation of development of Au-NP decorated ZnO–TiO_2_ core–shell NWs on the Si-wafer. Adapted with permission from ref. 102 Copyright (2018) American Chemical Society (ACS).

Park *et al.* demonstrated the development of Au-NP decorated ZnO–TiO_2_ core–shell NWs *via* a two-step process; the preparation of ZnO–TiO_2_ core–shell NWs was carried out using chemical vapor deposition (CVD), and decoration of Au-NPs was done on the ZnO–TiO_2_ core–shell NWs using photodeposition (PD).^102^ A schematic representation of ZnO, ZT, and ZTA NWs heterostructure synthesis is shown in Fig. 10. Primarily, a thin layer of carbon was coated on a Si-wafer. Then, well-aligned ZnO NWs were grown on the carbon-coated Si-wafer. Subsequently, a thin layer of TiO_2_ shell was deposited on the ZnO NWs core. Finally, Au-NPs were decorated on the ZnO–TiO_2_ core–shell NWs heterostructure by the PD method. It was observed that decoration of plasmonic Au-NPs enhanced the photocurrent density and photoconversion efficiency of as-prepared ZnO–TiO_2_ core–shell NWs due to increased visible light absorption, efficient electron extraction from Au-NPs due to the Schottky barrier, effective charge separation at multi-interface and transportation through 1D NWs. This study demonstrates that the plasmonic Au-NPs decorated on the ZnO–TiO_2_ core–shell NWs improve the PEC performance.

### 2.3. 2D structures

#### 2.3.1. TiO_2_ thin films

As discussed in the introduction, the particle geometry, size, and crystallinity of TiO_2_ are crucial to its photoactivity.^103–106^ The surface chemistry of TiO_2_ largely depends on the surface area, morphology, and exposed crystal planes.^105^ In general, the TiO_2_ NPs (P25) have been practically recognized as the “golden standard” photocatalyst because of their effectiveness in pollutant degradation (organic dyes and other pollutants) in water.^107^ However, TiO_2_ thin films are more suitable for water treatment and environmental remediation applications such as air purification.^108^ On the other hand, TiO_2_ thin films have a very inadequate surface area that hampers the effective photocatalytic degradation of organic pollutants.^109^ Hence, different strategies have been proposed to improve the volume-to-surface area ratio of TiO_2_ thin films for high photocatalytic activity. TiO_2_ thin films can be prepared by various methods such as physical vapor deposition (PVD) (including thermal evaporation, reactive sputtering, ion or electron beam evaporation), chemical vapor deposition (CVD) techniques, as well as wet chemical deposition methods such as sol–gel, solvothermal, dip-coating, spin-coating, and spray coating.^39,110–115^ As a factor of cost and ease of up-scaling, wet chemical methods are favored particularly for outdoor applications (self-cleaning textiles or exterior walls of buildings) of TiO_2_-based coatings. However, such methods usually require a secondary process, such as drying and high annealing temperature, to achieve stable and crystalline TiO_2_ layers, which is crucial for high photocatalytic performance.^116^

Alternatively, sputtering and evaporation techniques have been developed to produce columnar or sculptured thin films with enhanced porosity.^110^ Suzuki *et al.* reported that surface reaction enhanced the efficiency of obliquely deposited TiO_2_ thin films with different columnar geometrical shapes, including zigzag, cylindrical, and helical morphologies.^117^ More so, Goossens *et al.* reported a fractal (‘forest-like”) 3D TiO_2_ thin film.^118^ The choice of deposition method not only plays a role in the thin film structure but is also a contributing factor to the photocatalytic activity of the thin film.^119^ Another approach to enhance the photocatalytic activity of TiO_2_ thin films is the decoration by metallic nanostructures *via* photodeposition. The decoration of TiO_2_ thin films with noble metals such as Au, Ag, Pd, and Pt has been shown to exhibit excellent absorption properties due to the local surface plasmon resonance (LSPR) activities.^120^ Using Au and Ag for decorating TiO_2_ thin films has led to red-shifted and increased LSPR, which reduces charge recombination at the surface of TiO_2_, yielding an improved overall photocatalytic performance. Furthermore, those metal clusters are also electron acceptors and enhance charge separation, and there can be competing processes.

**Fig. 11:**
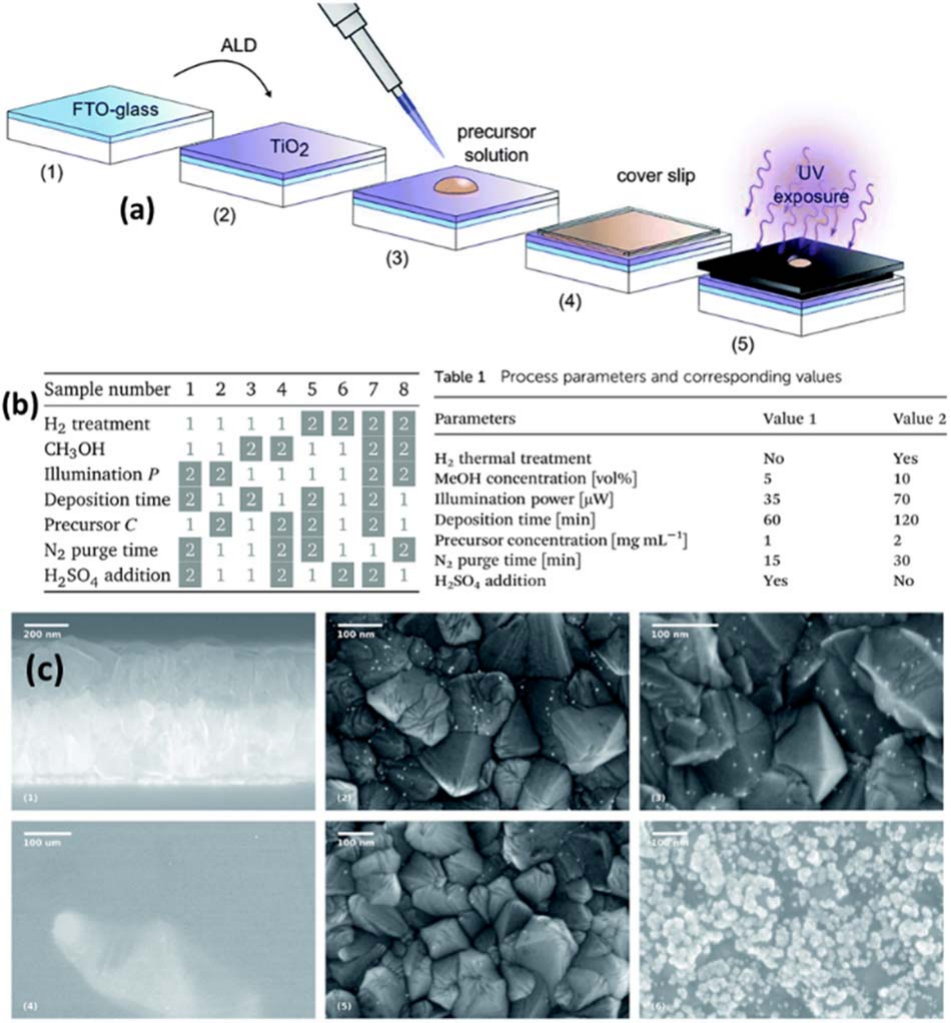
(a) Sample preparation and exposure: the H_2_ thermal treatment, when applied, takes place between steps (2) and (3). The illuminated area of the sample is 0.15 cm^2^ out of 5.5 cm^2^. (b) The eight combinations of parameters for each sample: the white boxes refer to value 1 in Table 1 (inset), while the gray boxes refer to value 2. (c) SEM micrographs: on the top, from left to right, are the section of the FTO-coated glass after the TiO_2_ deposition, and the surfaces of samples 5 and 8 after the platinum photodeposition, the nanoparticles shown in white. On the bottom part of the figure, three micrographs of sample 7 show the clear-colored platinum oxide well visible already at low magnification and higher magnifications of the darker area, where a few metallic particles are visible, and of the clear area, with the characteristic growth patterns for platinum oxide. Adapted with permission from ref. 121 (2019) Royal Society of Chemistry (RSC).

Tossi *et al.*^121^ explained the photodeposition of Pt NPs on TiO_2_ thin films that were grown *via* ALD (Fig. 11a). They mainly reported that the photodeposition parameters influence the control and prediction of the deposited nanoparticles' size, density, and loading. Fig. 11c summarizes the SEM images of different samples under different reaction conditions. On the top, the TiO_2_ deposited FTO-coated glass, samples 5 (see the corresponding parameters in Fig. 11b) and 8 (see the corresponding parameters in Fig. 11b) after the platinum photodeposition are shown with white parts as NPs. On the bottom, three SEM images of sample 7 (see the corresponding parameters in Fig. 11b) are given. They indicated that platinum oxide is visible even at low magnifications, and only a few metallic particles are visible at higher magnifications, which is a characteristic of platinum oxide. This work analyzed the photodeposition method for metal NPs from liquid precursors on ALD-grown films.

**Fig. 12:**
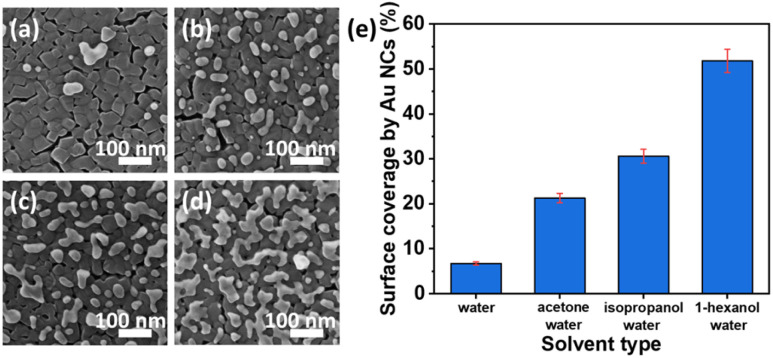
HIM images of Au NCs deposited onto TiO_2_ in (a) water, (b) acetone–water (v/v: 20/80), (c) isopropanol–water (v/v: 20/80), and (d) 1-hexanol–water (v/v: 20/80) mixtures. (e) Surface coverage (%) by Au NCs deposited with different solvents. Adapted with permission from ref. 122. Copyright (2020) American Chemical Society (ACS).

Recently, a highly active Au nanocluster (NC) fabrication on a TiO_2_ thin film surface by the photodeposition method was demonstrated.^122^ The surface coverage of TiO_2_ by Au NCs was found to be controlled by changing the solvent type and the illumination time. Higher photocatalytic performance was observed at a low surface coverage due to the high optical absorption of TiO_2_ at UV wavelengths. As indicated in morphological analysis (HIM images in Fig. 12a–d), different solvents (acetone, isopropanol, and 1-hexanol) were investigated to obtain the impacts on the photocatalytic depositions of Au NCs on TiO_2_. It was observed that water, among the other solvents, resulted in the lowest surface coverage value by Au NCs on TiO_2_ films. In contrast, the highest density of Au nanoclusters was achieved by a mixture of 1-hexanol–water as a solvent, as illustrated in Fig. 12e.

**Fig. 13:**
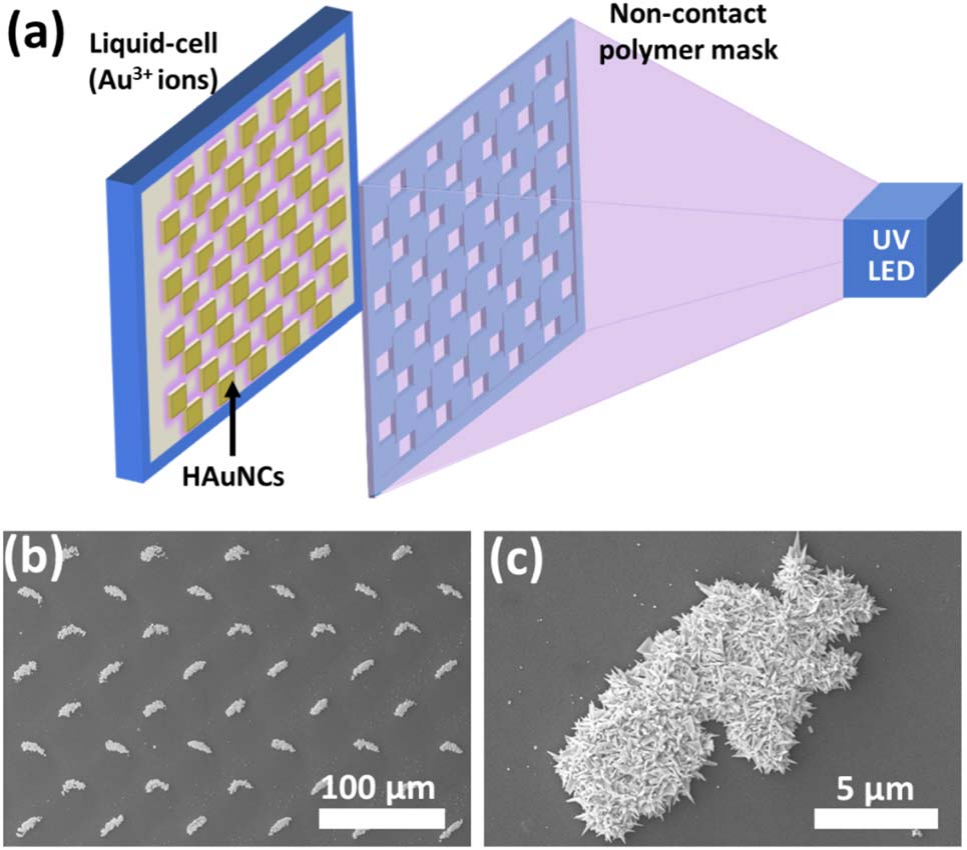
(a) Schematic representation of photocatalytic patterning of the TiO_2_ thin film with HAuNCs using a noncontact polymer mask. SEM images of (b) periodic HAuNC arrays and (c) a single HAuNC. Adapted with permission from ref. 20. Copyright (2018) Wiley.

As mentioned in the previous sections, the photocatalytic deposition method gives strong adhesion between deposited noble metals and the semiconductor surface. It is essential to use these hybrid nanostructures for various applications, especially for continuous water systems. The photocatalytic deposition approach can also pattern micro-/nanostructures without any stabilizer or surfactant, as shown in Fig. 13.^20^Fig. 13a illustrates the photocatalytic deposition of hierarchical Au needle clusters (HAuNCs) on a highly active TiO_2_ thin film surface. The non-contact polymer mask is located between the UV LED and the thin film sample. The TiO_2_ thin film surface can be selectively illuminated to reduce Au ions as metallic Au in only these illuminated areas (Fig. 13b and c). The rest remains empty due to UV LED non-illumination. The photocatalytic deposition approach is practical for preparing various shapes and patterned noble metal structures on semiconductors for well-desired applications (catalysis, plasmonic, and biomedical technologies).

## 3 Concluding remarks and future perspectives of photocatalytic deposition

In recent years, extensive research on noble metal–TiO_2_ nanostructures has demonstrated their crucial role in various applications such as water splitting, self-cleaning, CO_2_ reduction, and water remediation. The structure of the noble metal–TiO_2_ composite is critical in determining its photocatalytic properties. Various studies in the literature on preparing noble metals with different shapes and sizes exist. However, obtaining good adhesion between the noble metal and TiO_2_ surface is still challenging. Photodeposition is a promising approach to obtain well-desired noble metal structures on TiO_2_ surfaces with strong adhesion. Noble metal NPs can be quickly grown on the TiO_2_ surface under light exposure. However, numerous parameters (pH, temperature, precursor, electron sacrificial agent, *etc.*) affect the size and distribution of the deposited particles. In addition to these parameters, the interaction between solution and OD, 1D, and 2D morphologies might differ due to surface tension forces, drastically affecting the uniformity of the deposited metal nanostructures. This review focused on a few significant types of TiO_2_ morphologies (0D: NPs and nanocrystals; 1D: nanotubes and nanowires; and 2D: thin films) to better understand the photocatalytic deposition process. Based on available data, more fundamental research on different TiO_2_ morphologies is still needed to develop highly active photocatalytic structures for specific applications. Hopefully, this review can inspire multidisciplinary research interest in precisely tailoring TiO_2_ morphology to study in various applications.

## Data availability

No primary research results, software or code have been included and no new data were generated or analysed as part of this review.

## Conflicts of interest

There are no conflicts to declare.
